# Impact of Obesity and Ageing on the Expression of Key Mediators of SARS-CoV-2 Infection in Human Adipose Tissue

**DOI:** 10.3390/ijms26157313

**Published:** 2025-07-29

**Authors:** Maria Salazar, Mariana Ferreira, Sandra Marisa Oliveira, Francisca Saraiva, Carlos Pinho, Mariana Jarnalo, Inês Correia-Sá, Inês Falcão-Pires, Adelino Leite-Moreira, Delminda Neves, Henrique Almeida, Adriana R. Rodrigues, Alexandra M. Gouveia

**Affiliations:** 1 Unidade de Biologia Experimental, Departamento de Biomedicina, Faculdade de Medicina da Universidade do Porto, Alameda Prof. Hernâni Monteiro, 4200-319 Porto, Portugal; mariajsalazar88@gmail.com (M.S.); adrod@med.up.pt (A.R.R.); 2 i3S—Instituto de Investigação e Inovação em Saúde, Universidade do Porto/IBMC—Instituto de Biologia Molecular e Celular, Universidade do Porto, Rua Alfredo Allen 208, 4200-135 Porto, Portugal; 3 Cardiovascular R&D Centre—UnIC@RISE, Department of Surgery and Physiology, Faculty of Medicine, University of Porto, Alameda Prof. Hernâni Monteiro, 4200-319 Porto, Portugal; 4 Department of Plastic and Reconstructive Surgery, Instituto Português de Oncologia do Porto FG, Rua Dr. António Bernardino de Almeida, 4200-072 Porto, Portugal; 5 Department of Plastic, Reconstructive and Aesthetic Surgery, and Burn Unit, Centro Hospitalar e Universitário de São João, University of Porto, Alameda Prof. Hernâni Monteiro, 4200-319 Porto, Portugal; 6 Faculty of Nutrition and Food Sciences of the University of Porto, Rua do Campo Alegre 823, 4150-180 Porto, Portugal

**Keywords:** obesity, ageing, adipose tissue, COVID-19, SARS-CoV-2 receptors, ACE2, TMPRSS2, ADAM17, NRP1

## Abstract

Increased body mass index (BMI) and age are associated with COVID-19 severity. SARS-CoV-2 infection occurs through ACE2 binding, with TMPRSS2, ADAM17, and NRP1 facilitating this process. This study describes how adipose tissue (AT) location, BMI, age, and obesity affect these proteins’ expression. AT was collected from subcutaneous (abdominal superficial [AS], abdominal deep [AD], thigh [T]) and visceral (epiploon [E]) areas from middle-aged women without obesity (BMI 23.9 kg/m^2^, age 48.3 years). Subcutaneous AT was also obtained from middle-aged women with previous obesity (BMI 24.8 kg/m^2^, previously 41.7 kg/m^2^, age 46.9 years), older women with obesity (BMI 32.3 kg/m^2^, age 70.8 years), and older women without obesity (BMI 23.7 kg/m^2^, age 70.6 years). ACE2, TMPRSS2, ADAM17, and NRP1 expression was evaluated by qPCR and Western blotting. All proteins were more expressed in visceral AT. ACE2, TMPRSS2, and NRP1 positively correlated with BMI in AS and/or E, while NRP1 correlated with age in T. In subcutaneous AT, ACE2 and NRP1 were more influenced by obesity while TMPRSS2 was more age-dependent. In women with previous obesity, ACE2 and NRP1 levels decreased, while TMPRSS2 and ADAM17 remained unchanged. These findings highlight the differential influence of visceral AT, obesity, and age on the expression of SARS-CoV-2 cell entry mediators, potentially contributing to COVID-19 severity.

## 1. Introduction

The COVID-19 global pandemic, caused by SARS-CoV-2 virus infection, has emerged as a significant health concern. Several risk factors, such as age, hypertension, type 2 diabetes, and obesity are associated with COVID-19 severity [1,2,3,4]. Obesity is characterized by an excessive deposition of adipose tissue and is associated with several comorbidities, such as type 2 diabetes, hypertension, and heart diseases. According to the World Health Organization, in 2022, 2.5 billion adults were overweight and, of these, 890 million present with obesity. A body mass index (BMI) over 25 kg/m^2^ is considered overweight, while over 30 kg/m^2^ corresponds to obesity. Obesity induces a profound remodelling of the subcutaneous and visceral adipose tissue (AT), including an increase in inflammatory molecule secretion [5]. In fact, patients with obesity and COVID-19 present higher hospital admission rates, increased disease severity, often requiring mechanical ventilation, and higher mortality rates [6,7,8,9]. This outcome is the likely result of the compression of the diaphragm and lungs caused by abdominal obesity and/or to the chronic inflammation associated with obesity-induced adipose tissue (AT) dysfunction [10]. Another explanation relates to the SARS-CoV-2 virus’s high affinity for its main receptor, the human angiotensin-converting enzyme 2 (ACE2), a member of the renin–angiotensin system [11]. ACE2 is an 805 amino acid protein composed of an extracellular N-terminal domain to which SARS-CoV-2 binds, a transmembrane region, and a short intracellular C-terminal domain. This receptor is expressed in human AT in higher levels compared to the lung tissue [11,12,13], highlighting the importance of AT to SARS-CoV-2 infection. Moreover, the susceptibility of AT to SARS-CoV-2 infection depends on its anatomical location. In vitro, adipocytes from the visceral compartment present higher levels of ACE2 expression and infection rates compared to those from the subcutaneous depot [14]. It was reported that SARS-CoV-2 directly infects mature adipocytes and AT-resident macrophages [15,16].

SARS-CoV-2 infection is not only mediated by ACE2 as other proteins are also implicated in virus cell entry. The SARS-CoV-2 virus enters host cells through the binding of its viral spike proteins to cellular ACE2 receptors. The S protein must be primed by cell proteases, such as transmembrane protease serine 2 (TMPRSS2) and A disintegrin and metalloproteinase 17 (ADAM17) [17,18]. TMPRSS2 and ADAM17 also cleave the ACE2 receptor itself [19]. ADAM17 contributes to the shedding of ACE2 within amino acids 652 to 659 and the release of an extracellular soluble form of ACE2 [20], which binds to extracellular viral particles, preventing their entry into cells. TMPRSS2 sheds ACE2 within amino acids 697 to 716 to promote the fusion of SARS-CoV-2 with the host cell membrane [21]. In addition to the ACE2 receptor, neuropilin-1 (NRP1) was identified as a SARS-CoV-2 co-receptor [22]. It binds to the virus spike protein and, when co-expressed with ACE2 and TMPRSS2, potentiates SARS-CoV-2 infection [22,23].

Age-related changes in AT composition and function, such as impaired adipocyte progenitor function, increased senescent cells, and pro-inflammatory adipocytokine production [24], are also contributors to the severity of COVID-19.

Given the interplay between AT function, obesity, and ageing, it is critical to further investigate how these conditions influence the mechanisms of AT SARS-CoV-2 infection. Specifically, we aim to characterize the expression patterns of the key SARS-CoV-2 cell entry mediators ACE2, TMPRSS2, ADAM17, and NRP1 across distinct subcutaneous and visceral AT domains in order to understand their modulation by weight gain, ageing, and weight loss. These findings could have significant implications for understanding COVID-19 pathophysiology and for future provision of preventive actions and the development of targeted therapeutic strategies for high-risk populations, including individuals with obesity and advanced age.

## 2. Results

### 2.1. Anthropometric and Biochemical Characterization of All Individuals

To characterize the susceptibility of the different AT depots to SARS-CoV-2 entry and understand why obesity and ageing predispose to a higher risk of severe COVID-19, we examined AT from various patient groups. These groups were categorized according to BMI and age as follows: middle-aged women without obesity (control group, BMI 19.88–29.48 kg/m^2^, age 39–56 years); middle-aged women with previous obesity (BMI 22–27.2 kg/m^2^, previously 30.9–52.6 kg/m^2^, age 31–60 years); older women with obesity (BMI 30.43–35.52 kg/m^2^, age 61–81 years); and older women without obesity (BMI 19.56–26.30 kg/m^2^, age 62–81 years).

**Figure 1:**
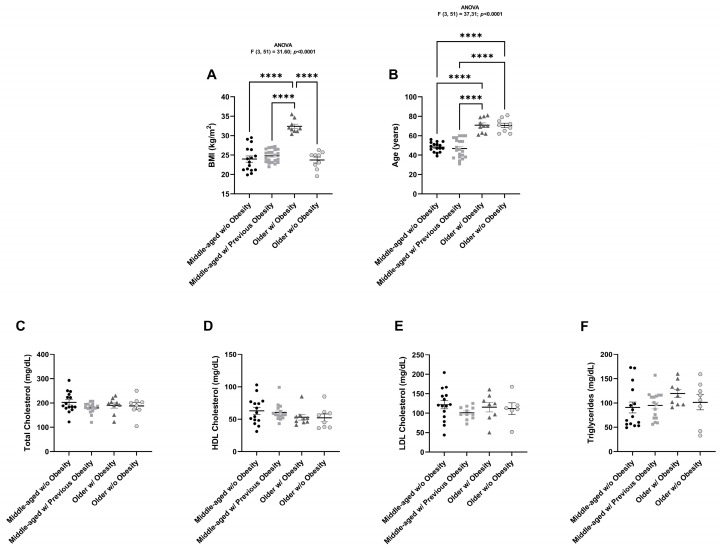
Graphical representation of the anthropometric and biochemical parameters from all women studied. BMI (kg/m^2^) (**A**), age (years) (**B**), total cholesterol levels (mg/dL) (**C**), HDL cholesterol levels (mg/dL) (**D**), LDL cholesterol levels (mg/dL) (**E**), and triglyceride levels (mg/dL) (**F**) of middle-aged women without obesity (n = 15–16), middle-aged women with previous obesity (n = 11–21), older women with obesity (n = 8–10) and older women without obesity (n = 6–9). The results are represented as mean ± S.E.M. **** *p* < 0.0001, according to the one-way ANOVA, followed by Tukey’s multiple comparisons test.

In terms of BMI, statistically significant differences (*p* < 0.0001) were observed between the group composed of women with obesity (32.26 kg/m^2^) and the control group (23.9 kg/m^2^), the group with previous obesity (24.8 kg/m^2^), and the older group without obesity (23.7 kg/m^2^) (Figure 1A). Age differs significantly (*p* < 0.0001) between the middle-aged groups (control group and group with previous obesity, with average ages of 48.3 and 46.9 years, respectively) and the older groups (the group with obesity and the group without obesity, with average ages of 70.8 and 70.6 years, respectively) (Figure 1B).

Regarding medical conditions that could potentially increase the risk of severe COVID-19, the group of middle-aged women without obesity had almost no comorbidities, except for four individuals with hypertension and one with type 2 diabetes. Similarly, in the group of women with previous obesity, only one presented with dyslipidaemia. In contrast, in the older groups, women presented with more comorbidities. The group with obesity includes ten women with dyslipidaemia, nine with hypertension, four with type 2 diabetes, and one with chronic lung disease. Among the older women without obesity, four had hypertension, five dyslipidaemia, one type 2 diabetes and three chronic lung disease. All patients were under appropriate medication to manage these conditions. As expected, women in the older groups were highly medicated; for example, almost all of them in both older groups, with or without obesity, were taking antihypertensive medication.

No differences were found in total cholesterol, HDL cholesterol, LDL cholesterol, and triglyceride levels among these groups (Figure 1C–F).

### 2.2. SARS-CoV-2 Cell Infection Mediators Have Increased Expression in Visceral Adipose Tissue of Middle-Aged Women Without Obesity

ACE-2, TMPRSS2, ADAM17, and NRP1 are the main cell membrane mediators that promote entrance of SARS-CoV-2 viruses into cells. To clarify if their expression pattern depends on the anatomical location of AT, the expression of these cell entry factors was evaluated in subcutaneous (AS, AD, and T) and visceral (E) compartments of AT from the middle-aged control patients.

**Figure 2:**
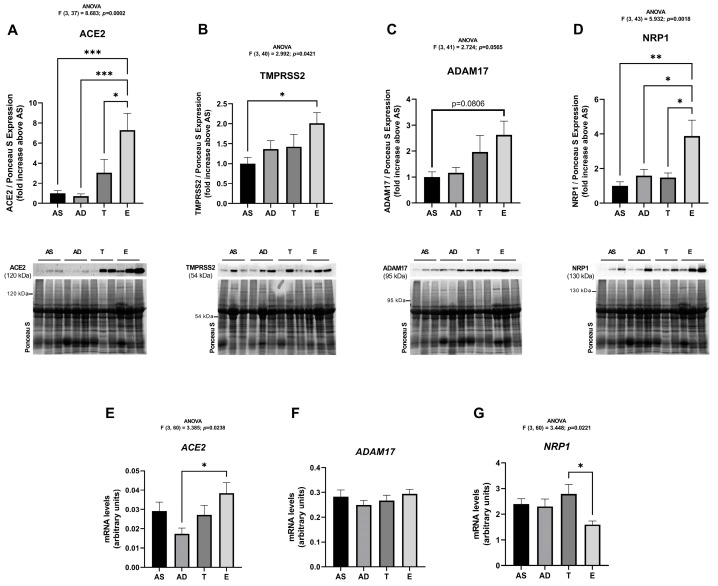
Expression of SARS-CoV-2 cell entry factors in human subcutaneous (AS—abdominal superficial; AD—abdominal deep; and T—thigh) and visceral (E—epiploon) adipose tissue from middle-aged individuals without obesity (BMI from 19.88 to 29.48 kg/m^2^), quantified by Western blot and real-time PCR. ACE2 (**A**), TMPRSS2 (**B**), ADAM17 (**C**), and NRP1 (**D**) protein expression in AS (n = 10–12), AD (n = 11–12), T (n = 10–12) and E (n = 9–12). ACE2 (**E**), ADAM17 (**F**), and NRP1 (**G**) mRNA expression in AS (n = 16), AD (n = 16), T (n = 16), and E (n = 16). The results are represented as mean ± S.E.M. * *p* < 0.05, ** *p* < 0.01, and *** *p* < 0.001, according to the one-way ANOVA, followed by Tukey’s multiple comparisons test.

ACE2 protein expression in epiploon AT (E) was 7-fold higher than in subcutaneous AS and AD (*p* = 0.0005 and 0.0003, respectively) and 2.3-fold higher (*p* = 0.0314) than in the T depot (Figure 2A). TMPRSS2 expression was 2-fold increased in the E depot relative to AS (*p* = 0.0259) (Figure 2B). ADAM17 levels were increased 2-fold in E when compared to AS (although this result is not statistically significant, it is very close to reaching significance; *p* = 0.0806) (Figure 2C). The NRP1 protein expression pattern parallels the one observed in ACE2: it is increased in E relative to all subcutaneous depots (AS: *p* = 0.002, AD: *p* = 0.0187 and T: *p* = 0.0154) (Figure 2D). Globally, the protein expression of these SARS-CoV-2 cell entry factors, evaluated by Western blot, was increased in visceral AT when compared with the subcutaneous counterparts (Figure 2). No differences were found within subcutaneous AT depots. A similar pattern was observed in the subcutaneous AT of patients with previous obesity, with no significant differences found between fat depots from the abdominal (A), thigh (T), and arm areas (Figure S1).

Regarding ACE2 mRNA expression, levels were higher in visceral tissue (E) when compared to the subcutaneous AD fraction (*p* = 0.0124) and no differences were obtained when comparing with AS and T AT samples (Figure 2E). ADAM17 mRNA is equally expressed in all fat tissues (Figure 2F). Surprisingly, the mRNA expression of NRP1 was decreased in E when compared to T (*p* = 0.0131) (Figure 2G), contrary to the results obtained at the protein expression level.

### 2.3. The Protein Levels of SARS-CoV-2 Cell Entry Mediators Positively Correlate with BMI

**Figure 3:**
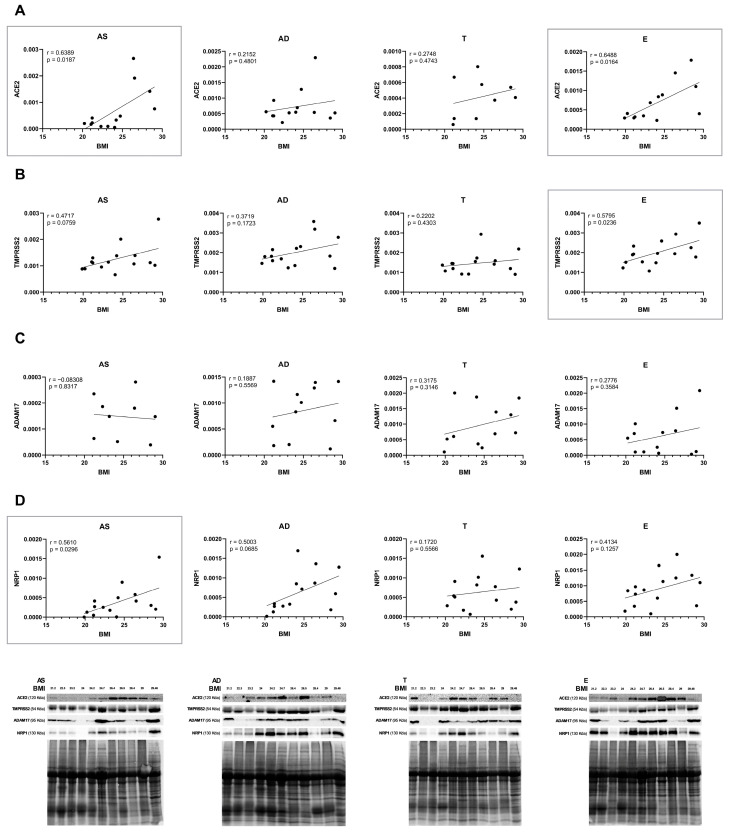
Correlations of SARS-CoV-2 receptor and co-receptor protein levels with BMI, quantified in human subcutaneous (AS—abdominal superficial; AD—abdominal deep; and T—thigh) and visceral (E—epiploon) adipose tissue from middle-aged individuals without obesity (BMI from 19.88 to 29.48 kg/m^2^) by Western blot. Respective representative blots are presented in the figure. ACE2 (**A**), TMPRSS2 (**B**), ADAM17 (**C**), and NRP1 (**D**) protein level correlation with BMI in AS (n = 9–15), AD (n = 12–15), T (n = 9–15), and E (n = 13–15). *p*-values lower than 0.05 are considered statistically significant, according to the Pearson correlation coefficient.

Within the control group, the protein expression levels of ACE2, TMPRSS2, ADAM17, and NRP1 in all AT compartments (AS, AD, T, and E) were correlated with the subject’s BMI. Generally, there is a tendency for a positive correlation, though statistically significant results are only observed for ACE2, TMPRSS2, and NRP1 in specific fat compartments, as highlighted in Figure 3. ACE2 protein levels positively correlate with BMI in AS and E depots (*p* = 0.0187 and 0.0164, respectively) (Figure 3A). Additionally, there is a statistically significant positive correlation in the protein expression of TMPRSS2 in E (*p* = 0.0236) and of NRP1 in AS (*p* = 0.0296) (Figure 3B,D) with BMI. Moreover, two correlations almost reached significance, the protein levels of TMPRSS2 in AS and NRP1 in AD (*p* = 0.0759 and *p* = 0.0685, respectively) (Figure 3B,D).

**Figure 4:**
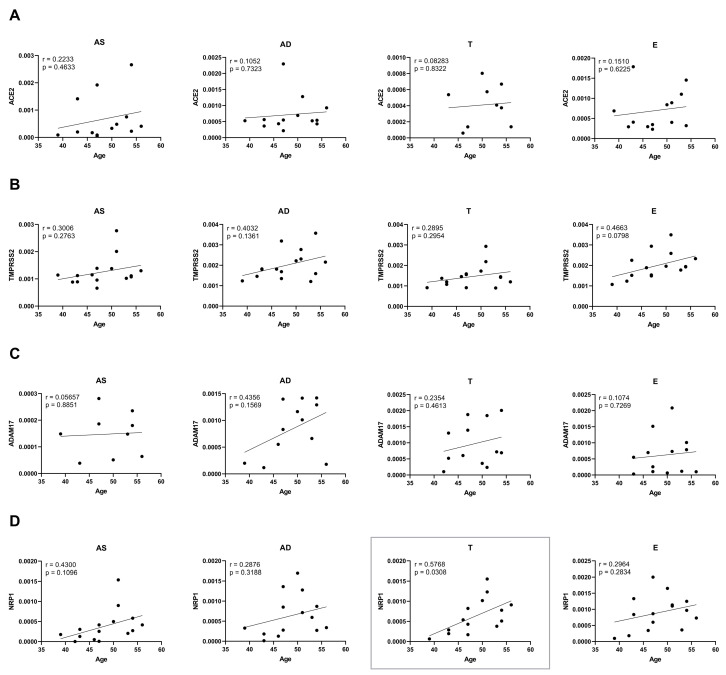
Correlations of SARS-CoV-2 receptor and co-receptor protein levels with age, quantified in human subcutaneous (AS—abdominal superficial; AD—abdominal deep; and T—thigh) and visceral (E—epiploon) adipose tissue from middle-aged individuals without obesity (BMI from 19.88 to 29.48 kg/m^2^) by Western blot. Representative blots are the same as in Figure 3. ACE2 (**A**), TMPRSS2 (**B**), ADAM17 (**C**), and NRP1 (**D**) protein level correlation with age in AS (n = 9–15), AD (n = 12–15), T (n = 9–15), and E (n = 13–15). *p*-values lower than 0.05 are considered statistically significant, according to the Pearson correlation coefficient.

Age is known to be an important factor in the modulation of SARS-CoV-2 infection [1]. To address this, correlations between the levels of all SARS-CoV-2 cell entry mediators and age were also determined in the control group (Figure 4). Although there is also a general tendency towards positive correlations, only NRP1 levels in the T depot significantly increase with ageing (*p* = 0.0308, Figure 4D). In conclusion, the expression of analysed receptors and coreceptors appears to positively correlate more prominently with BMI than with age.

**Figure 5:**
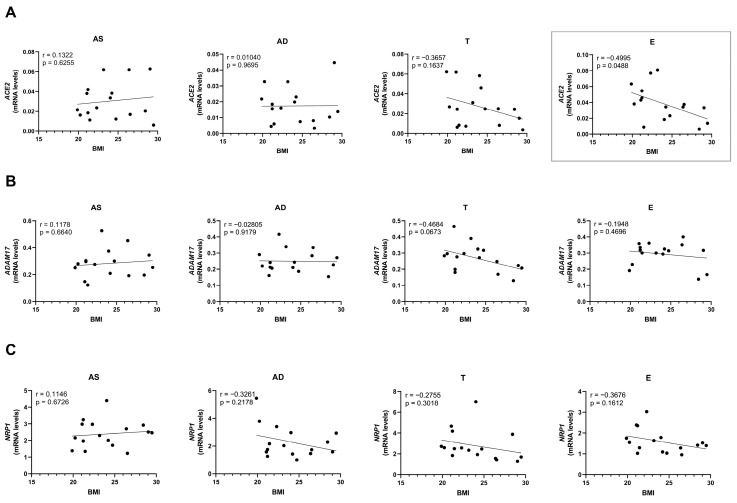
Correlation of SARS-CoV-2 receptor and co-receptor mRNA levels with BMI, quantified in human subcutaneous (AS—abdominal superficial; AD—abdominal deep; and T—thigh) and visceral (E—epiploon) adipose tissue from middle-aged individuals without obesity (BMI from 19.88 to 29.48 kg/m^2^) by real-time PCR. ACE2 (**A**), ADAM17 (**B**), and NRP1 (**C**) mRNA level correlation with BMI in AS (n = 16), AD (n = 16), T (n = 16), and E (n = 16). *p*-values lower than 0.05 are considered statistically significant, according to the Pearson correlation coefficient.

Unexpectedly, mRNA levels of ACE2 decrease with increasing BMI (*p* = 0.0488) in the E depot (Figure 5A), contrary to the protein levels, which showed a positive correlation with BMI (Figure 3A). No statistically significant differences were observed in mRNA expression of the other cell entry proteins evaluated. However, a tendency for mRNA expression to decrease with BMI in specific fat depots was noted.

### 2.4. Ageing and Obesity Differently Influence the Expression of the SARS-CoV-2 Cell Entry Factors in Subcutaneous Adipose Tissue

To further explore the combined effect of ageing and obesity on SARS-CoV-2 infection, the expression of ACE2, TMPRSS2, ADAM17, and NRP1 was evaluated in subcutaneous AT from older women with or without obesity. In addition, a group of samples collected from middle-aged patients with previous obesity was included to evaluate the effect of weight loss on the expression of the proteins.

**Figure 6:**
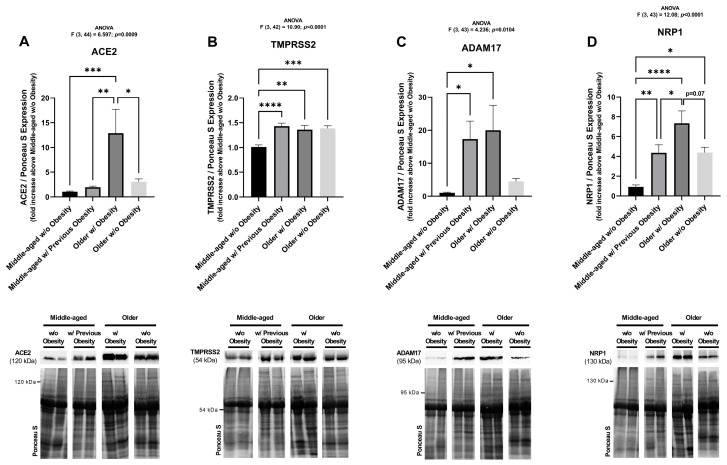
Expression of SARS-CoV-2 cell entry factors in human subcutaneous adipose tissue from middle-aged women without obesity (BMI from 19.88 to 29.48 kg/m^2^, average age of 48.2 years), middle-aged women with previous obesity (BMI from 22.0 to 27.2 kg/m^2^, previously 33.5 to 52.6 kg/m^2^, average age of 46.9 years), older women with obesity (BMI from 30.43 to 35.52 kg/m^2^, average age of 70.8 years), and older women without obesity (BMI from 19.56 to 26.3 kg/m^2^, average age of 70.6 years), quantified by Western blot. Representative blots are indicated in the figure. ACE2 (**A**), TMPRSS2 (**B**), ADAM17 (**C**), and NRP1 (**D**) protein expression in middle-aged women without obesity (n = 13–15), middle-aged women with previous obesity (n = 14), older women with obesity (n = 10) and older women without obesity (n = 9). The results are represented as mean ± S.E.M. * *p* < 0.05, ** *p* < 0.01, *** *p* < 0.001, and **** *p* < 0.0001, according to the one-way ANOVA, followed by Tukey’s multiple comparisons test.

In subcutaneous AT, older women with obesity have 4.2-fold higher ACE2 protein levels compared to older individuals without obesity (*p* = 0.0208) (Figure 6A). Thus, a clear effect of obesity on ACE2 receptor protein expression is observed in older patients. Moreover, in the group with previous obesity, the levels of ACE2 are significantly reduced compared to the group with obesity (*p* = 0.0028). In addition, ACE2 levels are significantly higher in older women with obesity when comparing to the middle-aged women without obesity (*p* = 0.001). Since no differences were detected between the middle-aged and older women without obesity, it is possible to conclude that the ageing effect on ACE2 expression is not as pronounced as obesity (Figure 6A).

Both TMPRSS2 and ADAM17 protein levels increase in older women with obesity, when compared to controls (*p* = 0.0013 and *p* = 0.0274, respectively), and do not differ significantly from middle-aged individuals with prior obesity (Figure 6B,C). TMPRSS2 levels increase with age (*p* = 0.0009, older group without obesity) to levels similar to those observed in obesity or after weight loss (Figure 6B).

Age alone induces a 4-fold increase in NRP1 protein levels (*p* = 0.0179 between control and older women without obesity groups). When advanced age is combined with obesity, those levels are almost twice as high. Although not statistically significant, they were close to reaching significance (*p* = 0.07). On the other hand, NRP1 protein levels decreased by almost half (*p* = 0.04) when comparing the groups of women with previous obesity and the older women with obesity, but still did not reach those observed in the control group, remaining elevated 4-fold and significantly higher (*p* = 0.0063) (Figure 6D).

Overall, the protein levels of all of the analysed SARS-CoV-2 cell entry factors are increased in older women with obesity (Figure 6) but these two factors (ageing and obesity) differently influence their expression.

## 3. Discussion

The present study highlights the importance of AT, specifically visceral AT, in the modulation of the clinical response to SARS-CoV-2 infection. In fact, it demonstrates that the expression of the SARS-CoV-2 cell entry mediators ACE2, TMRPSS2, ADAM17, and NRP1 is increased in human visceral AT. Accordingly, a computed tomography analysis of patients with symptomatic COVID-19 found an association between higher visceral AT area and severe forms of COVID-19, whereas subcutaneous fat levels were not related [25]. Moreover, Saccon et al. found increased expression of ACE2 receptor in primary cultured visceral adipocytes, which were more prone to SARS-CoV-2 infection compared with the subcutaneous [14]. Both TMPRSS2 and ADAM17 cleave ACE2 and viral S protein to facilitate SARS-CoV-2 fusion with host cell membranes [17,26], promoting a crucial environment conducive to viral entry and replication [17,27]. Conversely, ADAM17 may have a protective role against COVID-19 infection by releasing a soluble ACE2 isoform that binds to circulating viral particles, preventing their entry into host cells [20]. NRP1 is expressed in macrophages [28], more abundant in human visceral than in subcutaneous AT [29]. This supports the current findings of higher NRP1 protein expression in the visceral depot. NRP1 is also involved in immune function, which is particularly relevant given the exaggerated immune responses associated with severe COVID-19 [30]. Therefore, the increased levels of SARS-CoV-2 cell entry factors in the visceral adipose tissue suggests that this depot may act as a reservoir for viral particles, potentially contributing to systemic viral spread that ultimately leads to the disruption in immune signalling and severe inflammatory responses observed in COVID-19 patients with a higher volume of visceral fat [25].

Obesity, a condition accompanied by increased serum ACE2 levels [31], and excess weight are known risk factors for severe forms of COVID-19 [6,32,33]. This is supported by the positive correlation between the protein expression levels of ACE2, TMPRSS2, and NRP1 and BMI, both in subcutaneous and visceral AT, herein described. Similar findings have been reported: Patel et al. described increased ACE2 protein levels in epicardial AT of patients with obesity and diet-induced obese mice [34]; higher ACE2 gene expression was reported in epicardial and subcutaneous AT of individuals with obesity [35]; and Frühbeck and colleagues showed that ACE2 mRNA increases only in visceral and not in the subcutaneous depot of patients with obesity [36]. Only a single, sample-limited immunofluorescence study reported a lack of variation in ACE2 protein levels in human subcutaneous AT from lean or obese individuals [37]. TMPRSS2 gene expression was found to be increased in lung epithelial cells and in pancreatic islets of subjects with obesity [38,39]. As well, rats fed a high fat and sugar diet also present elevated protein expression of TMPRSS2 in the lungs compared with the controls [40]. NRP1 mRNA expression significantly increases in the subcutaneous AT of patients with obesity and type 2 diabetes. In patients with obesity but without type 2 diabetes, its levels also increase, although without statistical significance [36], as shown in the current work. Conversely, in immunofluorescence studies, Steenblock et al. found no differences in NRP1 protein expression in subcutaneous AT between individuals with or without obesity [37]. The different methodologies implied for protein quantification (Western blotting versus immunofluorescence) may justify the divergency in results.

Interestingly, here, a different pattern of expression of the receptors (ACE2 and NRP1) and co-receptors (ADAM17 and TMRPSS2) with obesity and ageing is reported. In older individuals without obesity, protein levels of subcutaneous ACE2 and NRP1 receptors, to which SARS-CoV-2 binds directly, did not increase as markedly as they did in individuals of the same age with obesity. Thus, an evident effect of obesity in modulation of ACE2 and NPR1 expression was demonstrated. The ageing effect on the expression of these proteins is controversial in the literature since some studies report no correlation of ACE2 and NRP1 gene expression with age in subcutaneous AT [41,42] but in a gene set database analysis, ACE2 gene expression was found to be increased in older individuals [43].

ADAM17 expression in subcutaneous AT seems to depend simultaneously on ageing and obesity, since the isolated factors did not significantly influence ADAM17 protein levels. Dou et al. found that age and obesity are associated with increased ADAM17 enzyme activity in human mediastinal AT blood vessels and adipocytes [44], which aligns with the present data, showing an increase in ADAM17 protein expression in subcutaneous AT from older individuals with obesity, a condition that parallels the enhanced expression of ADAM17 mRNA in the subcutaneous AT of obese mice [45]. In omental AT of patients with morbid obesity, ADAM17 is considered a predictor of insulin resistance [46]. Moreover, transgenic mice with ADAM17 overexpression presented increased macrophage infiltration and fibrosis when fed an HFD [47], whereas ADAM17 gene silencing in visceral macrophages improved AT inflammation and type 2 diabetes [48].

In turn, TMPRSS2 expression appears to be more dependent on ageing rather than obesity, since its expression remains virtually the same in both older groups (with or without obesity), while it increases in comparison to the middle-aged control group. Moreover, the expression of TMPRSS2 and ADAM17 remains unchanged in individuals with previous obesity. Since these are two proteases that are involved in cancer and inflammation processes [49,50], and subcutaneous AT from individuals with prior obesity retains molecular characteristics of obese AT [51], this may explain why there is no reversion in TMRPSS2 and ADAM17 receptor protein levels after weight loss. Patients with previous obesity, after undergoing bariatric surgery and stabilizing their weight for one year, underwent abdominoplasty, during which adipose tissue samples were collected. It is, however, unclear if, over a longer period, these patients would reach the levels of TMPRSS2 and ADAM17 observed in individuals without obesity. Conversely, the protein expression levels of ACE2 and NRP1, receptors to which SARS-CoV-2 directly binds, decrease in individuals with previous obesity. Other studies that access gene expression are in line with these findings; ACE2 levels have been shown to decrease in subcutaneous AT two years after bariatric surgery [52] and also after weight loss without surgical interventions [38,41]. Cauwenberghs et al. showed a decrease in the soluble form of ACE2, through proteomic measurements in human plasma, after 6 months of a restrictive diet [53], and Soll and colleagues described a decreased NRP1 mRNA expression in subcutaneous AT after weight loss [42].

The importance of the study of the diverse cell entry factors herein described is highlighted by the evolving infection behaviour of the SARS-CoV-2 variants [54,55,56]. It is known that when ACE2 levels are low, some co-factors, like ADAM9, promote SARS-CoV-2 infection, particularly in alpha (B.1.1.7), delta (B.1.617.2), and omicron (B.1.1.529) variants [57]. In fact, ADAM9 and other proteases, such as ADAM17 and TMPRSS2, interact with the spike protein to facilitate virus entry, especially in cells with limited ACE2 expression [57,58]. Additionally, new SARS-CoV-2 lineages such as KP.2, KP.3, and LB.1, although still dependent on ACE2, have subtle differences in receptor use in different species and tissues [59]. As shown herein, the concomitant expression of TMPRSS2, ADAM17, and NRP1 in adipose tissue—receptors that become elevated in obesity and, in the case of TMPRSS2 and ADAM17, remain elevated after weight loss—likely amplifies SARS-CoV-2 tropism and infectivity in adipose tissue, even when ACE2 levels decrease following weight loss. This observation is consistent with recent findings by Li et al., who demonstrated that in women with gestational diabetes, metabolic memory can sustain increased SARS-CoV-2 susceptibility, as metabolic alterations persist postpartum along with sustained low-grade inflammation [60]. Moreover, AT itself preserves an obesity-related epigenetic memory after weight loss [61]. This supports the notion that the persistent expression of these proteases observed in the adipose tissue samples after weight loss may reflect a broader metabolic memory effect, potentially relevant across different tissues and physiological contexts.

In this work, it was consistently observed that mRNA data did not align with the protein expression results. In accordance, Wang and colleagues found significant discrepancies between mRNA and protein levels of ACE2 within and throughout tissues when analysing proteomic and RNA-seq datasets [62]. These authors concluded that protein levels provide a more precise measurement of ACE2 abundance compared to mRNA levels. Regarding NRP1, an increase in its protein levels was observed in visceral fat compared to subcutaneous depots. Again, the opposite was observed in NRP1 mRNA levels, as already reported by Saccon et al. [14]. The regulation of NRP1 involves complex interactions with various proteins and microRNAs. Studies on breast and gastric cancer have shown that RNA binding proteins such as PUM2 and Lin28B can bind to NRP1 mRNA, increasing its stability and expression, thereby affecting protein levels [63,64]. Conversely, in glioblastoma and breast cancer studies, NRP1 is targeted by miR-124-3p and miR-376a, which suppress its expression [65,66]. These mechanisms illustrate the complex regulation of NRP1 at both mRNA and protein levels and suggest that a particular regulatory mechanism exists in AT. Apart from all of the mentioned interactions, other post-transcriptional mechanisms such as alternative splicing, RNA editing, translation efficiency, and protein degradation [67] could also contribute to the discrepancies between mRNA and protein levels observed in this study, which reinforces that protein abundance should be assessed to better reflect functional effects.

This study has some limitations on the retrospective nature of data collection and the homogeneity in the types of AT samples collected across all groups, much on account of the nature of the surgeries performed. Considering this, all adipose tissue samples were collected from women and, for this reason, the results obtained could be gender specific. The inclusion of adipose tissue samples from men would be important to the generalization of the conclusions.

Although the interference of comorbidities and medications with ACE2 expression is controversial [68,69,70,71,72,73], in the present study, consistent differences in the protein levels of the SARS-CoV-2 mediators were observed between groups with or without obesity, despite comparable comorbidity profiles and similar patterns of medication use. Therefore, the observed variations in ACE2, ADAM17, and NRP1 expression are primarily attributable to obesity itself rather than to confounding effects from underlying conditions or treatments.

## 4. Materials and Methods

### 4.1. Human Adipose Tissue Samples

Control AT samples were collected from 16 middle-aged women without obesity undergoing breast reconstruction surgeries after mastectomy, in the context of breast cancer, at the Instituto Português de Oncologia do Porto (IPO-Porto), from 4 different anatomical locations: subcutaneous abdominal superficial (AS), abdominal deep (AD), thigh (T), and visceral epiploon (E). At Centro Hospitalar Universitário São João (CHUSJ), subcutaneous AT samples were collected from 30 middle-aged women, with previous obesity, that underwent bariatric surgery and were subsequently referred to abdominoplasty, arm, and thigh lift surgeries. Samples were collected from the patient’s abdominal (A), arm, and thigh (T) regions. Subcutaneous AT from 10 older women with obesity and 9 older women without obesity were collected during cardiac surgeries (coronary, valvular, and ascending aorta) at the Cardiothoracic Surgery Service of CHUSJ. Those were harvested from the pre-sternal region. All samples were stored at −80 °C for subsequent protein and RNA extraction.

The patients’ age, BMI, cholesterol (total, HDL, and LDL) levels, and triglyceride levels were obtained by consulting the biochemical analysis performed nearest the time of respective surgery, when the tissues were collected. Unfortunately, it was not possible to have access to data on specific markers of inflammation.

**Figure 7:**
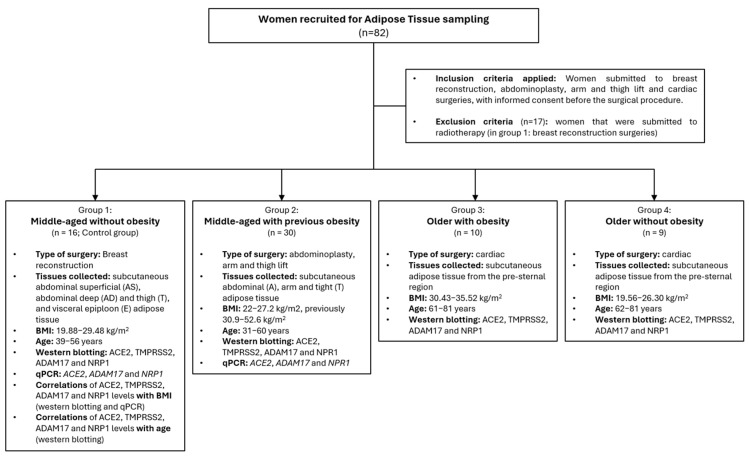
Flow chart summarizing all groups of patients recruited to this study divided according to age and obesity status. Inclusion/exclusion criteria, type of surgeries and respective AT anatomical location are also indicated in the figure.

A flow chart summarizing the inclusion criteria, established groups, sample collection sites, and analysis performed is presented in Figure 7.

### 4.2. Real-Time PCR

For RNA extraction, the TripleXtractor directRNA kit was used (#GK23.0100, Grisp, Porto, Portugal). A total of 100 to 200 mg of AT was weighed and placed in tubes with magnetic BulkBeads (Precellys, Montigny-le-Bretonneux, France) and homogenised in 700 μL of TripleXtractor reagent in a MagNA Lyser instrument (Roche, Mannheim, Germany) at 6500 rpm for 30 s. Then, samples were centrifuged at 15,000× *g* for 5 min at room temperature to remove the upper fat layer. RNA was extracted from the supernatant, following the RNA extraction kit manufacturer’s instructions. RNA concentration and purity were analysed using NanoDrop One equipment (ThermoFisher Scientific, Waltham, MA, USA). Then, agarose gel electrophoresis was performed to verify the integrity of the RNAs. A total of 300 ng of each RNA sample was run on a 1% agarose gel for 15 to 20 min at 100 mA. The 28S and 18S rRNA bands were visualized on a Chemidoc UV transilluminator (Chemi-DocTM XRS, Bio-Rad, Hercules, CA, USA). cDNA was then obtained from 400 ng of RNA using the NZY First-Strand cDNA Synthesis Kit (MB12501, Nzytech, Lisboa, Portugal), following the manufacturer’s instructions. Finally, real-time PCR was performed with the Power SYBR Green PCR Master Mix (ThermoFisher Scientific, Waltham, MA, USA). For each reaction, together with the SYBR Green mix, 10 ng of cDNA and 0.06 pmol of each primer (forward and reverse) were combined. Two thermocyclers were used, one for 96-well plates, the QuantStudio™ 3 Real-Time PCR System, 96-well, 0.1 mL Block (ThermoFisher Scientific, Waltham, MA, USA), and another for 384-well plates, the Real-Time PCR System (Bio-Rad, Hercules, CA, USA). The program was as follows: 10 min of denaturation at 95 °C, followed by 45 cycles of 10 s of denaturation at 95 °C, 30 s of annealing at 60 °C, and 30 s of extension at 60 °C. The expression of ACE2 (forward sequence: 5′-CATTGGTCTTCTGTCACCCGA-3′ and reverse sequence: 5′-CCCCAACTATCTCTCGCTTCAT-3′), ADAM17 (forward sequence: 5′-GGGCAGAGGGGAAGAGAGTA-3′ and reverse sequence: 5′-TGTGGAGACTTGAGAATGCGA-3′), and NRP1 (forward sequence: 5′-GCCACAGTGGAACAGGTGAT-3′ and reverse sequence: 5′-ATGACCGTGGGCTTTTCTGT-3′) genes was evaluated. Importin 8 (IPO8) was used as a reference gene (forward sequence: 5′-CCAAGGGGTGGTTCATTCT-3′ and reverse sequence: 5′-TCTTGCCACAGCTCTTCATC-3′). Control reactions without reverse transcriptase were performed to exclude non-specific amplification of genomic DNA.

### 4.3. Western Blotting

Total proteins were extracted from 100 to 200 mg of AT with lysis buffer (50 mM Tris pH 7.6, 10 mM NaCl, 5 mM EDTA, 1 mM β-glycerophosphate, and 0.25% *v*/*v* Triton X-100) supplemented with protease (1:500) and phosphatase (1:250) inhibitors (Sigma-Aldrich, St. Louis, MO, USA). Lysis of AT was performed in a MagNA Lyser Instrument (Roche, Mannheim, Germany), with magnetic BulkBeads (Precellys, Montigny-le-Bretonneux, France). Then, samples were sonicated for 15 min in the BioruptorTM UCD-200 (Diagenode, Liège, Belgium) and centrifuged at 10,000× *g* for 10 min at 4 °C to discard the fat layers. The supernatant was stored at −20 °C for later protein quantification by the Bradford Reagent assay (Bio-Rad, Hercules, CA, USA). A total of 30 μg of protein was loaded per well and resolved in 10% SDS-PAGE gels (25 mA per gel). Proteins were then transferred into nitrocellulose membranes (Bio-Rad, Hercules, CA, USA) for 2 h at 30 V. All membranes were stained with Ponceau S and one image of each was recorded in a Chemi-DocTM XRS instrument (Bio-Rad, Hercules, CA, USA). Then, membranes were blocked with 5% *w*/*v* non-fat dry milk (Molico^®^) dissolved in 0.1 M Tris-buffered saline with 0.1% Tween 20 (TBST), except the ones that were incubated with anti-TMPRSS2 antibody, that were blocked with 5% *w*/*v* bovine serum albumin (BSA) in TBST. After this, membranes were probed with primary antibodies (dissolved in 5% BSA, 0.1% NaN3 in TBST) specific for ACE2 (1:1000, abcam 108252, Cambridge, United Kingdom), TMPRSS2 (1:1000, abcam 109131, Cambridge, United Kingdom), ADAM17 (1:000, Santa Cruz Biotechnology sc-390859, Dallas, TX, USA), and NRP1 (1:1000, abcam 81321, Cambridge, United Kingdom), for three nights for ACE2 and one night for the remaining antibodies, at 4 °C. Subsequently, membranes were washed with TBST and incubated with the respective anti-rabbit (1:10,000, A16096, Invitrogen, Waltham, MA, EUA) or anti-mouse (1:5000, 715-035-150, Jackson Immuno Research, West Grove, PA, USA) horseradish-conjugated secondary antibodies diluted in 5% non-fat dry milk or 5% BSA (for TMPRSS2) in TBST for 1 h at room temperature with stirring. Finally, after washing the membranes with TBST, proteins were incubated with the Clarity ECL Western blotting substrates (Bio-Rad, Hercules, CA, USA). ECL signals and Ponceau S-stained membranes were captured in the Chemi-DocTM XRS instrument (Bio-Rad, Hercules, CA, USA) and quantified by densiometric analysis with the Image Lab 6.1 software (Bio-Rad, Hercules, CA, USA). Protein values were normalised by determination of the ratio of each ECL signal with the respective Ponceau S lane quantification. A Western blot analysis of internal loading controls, such as tubulin, actin, or Lamin A/C expression, was also performed. However, their protein expression levels linearly correlated with BMI, compromising their suitability as stable loading controls (Figure S2). This observation is consistent with previous reports [74,75]. Therefore, total protein staining with Ponceau S was used as protein loading normaliser, which is a widely accepted alternative when stable internal controls are not available [76].

Some membranes were re-used for incubation with other antibodies. For this, a 20 min stripping protocol with warm 10% SDS was performed, followed by washes with TBST, blocking, and incubation with specific primary and secondary antibodies.

### 4.4. Statistical Analysis

All statistical analyses were conducted in GraphPad Prism 9 (version 9.5.1). The correlation experiments were analysed by the Pearson correlation coefficient. The remaining analyses were performed using one-way ANOVA, followed by post-hoc analyses using Tukey’s multiple comparisons test. All results are presented as mean ± standard error of the mean (SEM) and *p*-values < 0.05 were considered statistically significant. Outliers were removed after identification by the ROUT method test using the GraphPad Software, setting Q to 1.0%, in the correlation experiments.

## 5. Conclusions

In conclusion, this study provides novel insights into the expression patterns of SARS-CoV-2 cell entry mediators in AT under conditions of obesity, ex-obesity, or advanced age. ACE2, TMPRSS2, ADAM17, and NRP1 are more expressed in visceral AT, with obesity significantly influencing their protein expression. This may enhance the susceptibility to SARS-CoV-2 infection and contribute to the severe inflammatory responses observed in COVID-19. Although ageing also contributes to increased expression of the receptors and co-receptors in AT, ACE2 and NRP1 expression is much more upregulated in obesity. Importantly, persistent TMPRSS2 and ADAM17 expression was shown, even after significant weight loss, reflecting the long-lasting impact of obesity on adipose tissue biology. Nevertheless, normal weight individuals with previous obesity may have lower COVID-19 risk since present reduced ACE2 and NRP1 protein levels, receptors that bind directly to the SARS-CoV-2 virus. These results underscore the critical need to continue to investigate the molecular mechanisms driving the expression of SARS-CoV-2 receptors and their implications for metabolic health and infectious disease susceptibility. Such efforts could ultimately lead to targeted therapeutic strategies aimed at mitigating the impact of adipose tissue and obesity on COVID-19 outcomes and broader metabolic complications.

## Data Availability

The data are not publicly available due to privacy and ethical restrictions but are available from the corresponding author by reasonable request.

## Supplementary Materials

The following supporting information can be downloaded at https://www.mdpi.com/article/10.3390/ijms26157313/s1.

## Abbreviations

The following abbreviations are used in this manuscript:

ACE2	Angiotensin-converting enzyme 2
AD	Abdominal deep adipose tissue
ADAM17	Protease A disintegrin and metalloproteinase 17
AS	Abdominal superficial adipose tissue
AT	Adipose tissue
BMI	Body mass index
CHUSJ	Centro Hospitalar Universitário São João
COVID-19	Coronavirus disease 2019
E	Epiploon adipose tissue
HDL	High-density lipoprotein
IPO-Porto	Instituto Português de Oncologia do Porto
IPO8	Importin 8
LDL	Low-density lipoprotein
NRP1	Neuropilin-1
S	Spike
SARS-CoV-2	Severe acute respiratory syndrome coronavirus 2
T	Thigh
TMPRSS2	Transmembrane protease serine 2
