# Supplementary material for: Impact of Obesity and Ageing on the Expression of Key Mediators of SARS-CoV-2 Infection in Human Adipose Tissue
Source: Int J Mol Sci. 2025 Jul 29;26(15):7313. doi: 10.3390/ijms26157313 (PMC12347392; doi:10.3390/ijms26157313)
Supplement: Supplementary file 1 [file ijms-26-07313-s001.zip › ijms-3703391-supplementary.pdf]

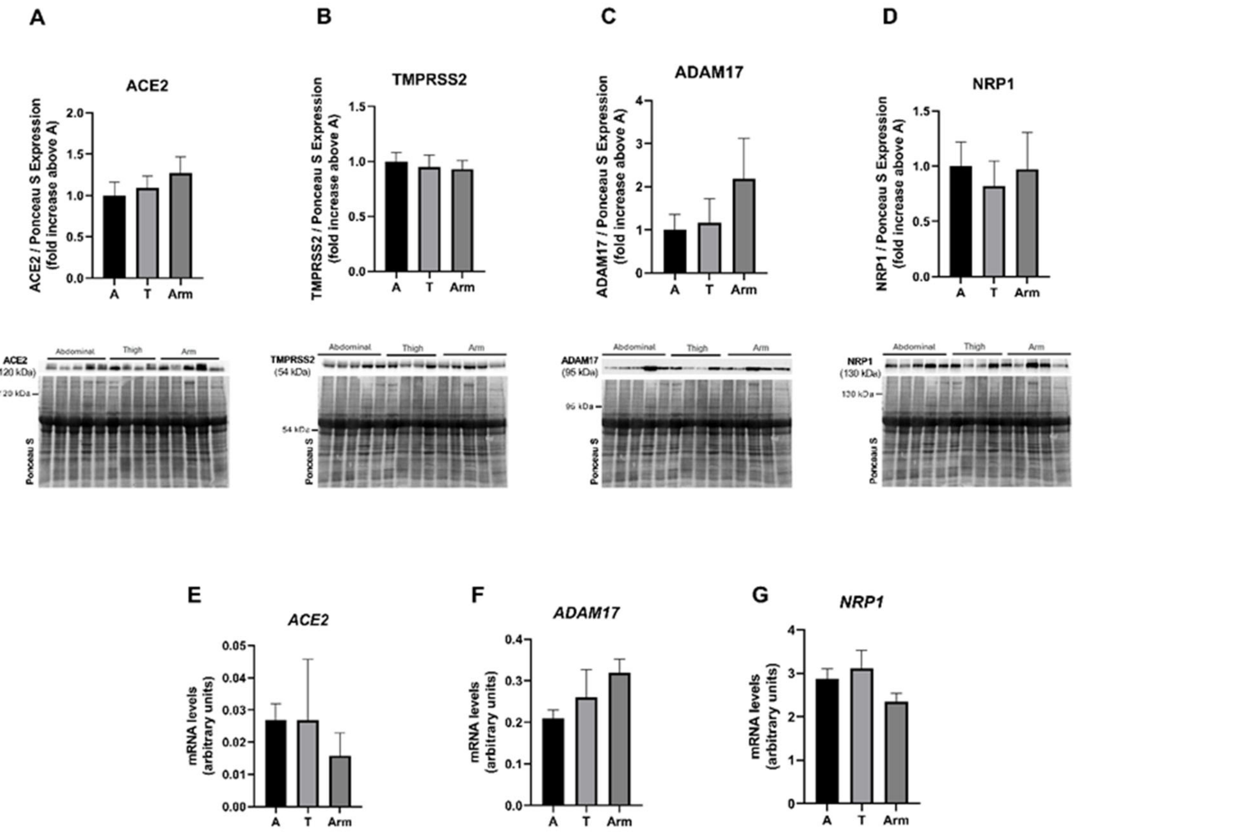

Figure S1. Expression of SARS-CoV-2 receptors and co-receptors in human subcutaneous adipose tissue (A—Abdominal, T—Thigh and Arm regions) from middle-aged patients with previous obesity (BMI from 22.0 to 27.2 kg/m^2, previously 33.5 to 52.6 kg/m^2), quantified by western blot and real-time PCR. ACE2 (**A**), TMPRSS2 (**B**), ADAM17 (**C**) and NRP1 (**D**) protein expression in A (n = 4–5), T (n = 4) and Arm (n = 5). ACE2 (**E**), ADAM17 (**F**) and NRP1 (**G**) mRNA levels in A (n = 22–24), T (n = 4) and Arm (n = 5) regions. The results are represented as mean ± S.E.M. and were analysed according to the One-Way ANOVA.

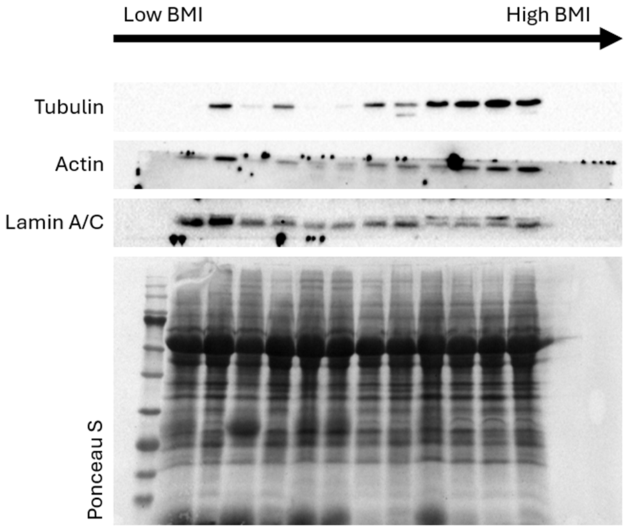

Figure S2. Western-blotting analysis of housekeeping proteins: Tubulin (Santa Cruz sc-32293), Actin (Santa Cruz sc-1616) and Lamin A/C (Santa Cruz sc-6215). The respective nitrocellulose membrane stained with Ponceau S is also shown. BMI values range from 19 to 29 kg/m^2.
